# Stroke-Like Episodes in PMM2-CDG: When the Lack of Other Evidence Is the Only Evidence

**DOI:** 10.3389/fped.2021.717864

**Published:** 2021-10-11

**Authors:** Mercedes Serrano

**Affiliations:** ^1^Department of Neuropediatric, Institut de Recerca Hospital Sant Joan de Déu, Barcelona, Spain; ^2^U-703 Center for Biomedical Research on Rare Diseases (CIBER-ER), Hospital Sant Joan de Déu, Instituto de Salud Carlos III, Barcelona, Spain

**Keywords:** channelopathies, congenital disorders of glycosylation, *CACNA1A*, PMM2-CDG, stroke-like, coma

## Abstract

Phosphomannomutase 2 deficiency (PMM2-CDG) is the most frequent congenital disorder of glycosylation. PMM2-CDG patients develop chronic cerebellar atrophy as a neurological hallmark. However, other acute neurological phenomena such as stroke-like episodes (SLE), epilepsy, migraine, and cerebrovascular events, may also occur, and they are frequently the cause of disability and impaired quality of life. Among these, SLE are among the most stressful situations for families and doctors, as their risk factors are not known, their underlying pathomechanisms remain undiscovered, and clinical guidelines for diagnosis, prevention, and treatment are lacking. In this paper, the recent SLE experiences of two PMM2-CDG patients are examined to provide clinical clues to help improve diagnosis through a clinical constellation of symptoms and a clinical definition, but also to support a neuroelectrical hypothesis as an underlying mechanism. An up-to-date literature review will help to identify evidence-based and non-evidence-based management recommendations. Presently neuropediatricians and neurologists are not capable of diagnosing stroke-like episodes in an unequivocal way, so there is still a need to perform invasive studies (to rule out other acute diseases) that may, in the end, prove unnecessary or even harmful. However, reaching a correct and early diagnosis would lead not only to avoidance of invasive tests but also to better recognition, management, and understanding of the disease itself. There is a great need for understanding of SLE that may ultimately be very informative for the detection of patients at risk, and the future development of preventive and management measures.

## Introduction

Congenital disorders of glycosylation (CDG) are a rapidly expanding group of metabolic disorders with defects in the synthesis of glycans and their attachment to proteins and lipids. To date, over 150 CDG types have been described (1). PMM2-CDG (MIM# 212065), caused by *PMM2* mutations, is by far the most frequent congenital disorder of N-linked glycosylation, with more than 900 patients in the literature (2).

PMM2-CDG patients develop cerebellar atrophy and a chronic cerebellar syndrome (3, 4). However, other acute neurological phenomena such as stroke-like episodes (SLE), epilepsy, migraine, movement disorders, and cerebrovascular events, may also occur (5–13), and they are frequently the cause of disability and impaired quality of life. Among these, SLEs are particularly stressful for families and doctors, as their risk factors are not known, their underlying pathomechanisms remain undiscovered, and clinical guidelines for diagnosis, prevention and treatment are lacking (5–12).

The term SLE was initially coined for MELAS syndrome to stress its non-ischemic origin (14) but it has been used for different neurological diseases associated with focal deficits that mimick, clinically but not neuroradiologically, an ischemic injury. This is the case of PMM2-CDG and some channelopathies related to *CACNA1A, ATP1A2*, and *SCN1A* mutations, leading to episodes called familial hemiplegic migraine (FHM). FHM is, actually, very similar clinically to SLE, as episodes are triggered by metabolic stress or cranial trauma, they present after a symptom-free period, and they progress to irritability, somnolence, focal deficits with hyperpyrexia, and, frequently epileptic seizures, eventually ending in clinical resolution after a period of time from hours to days (8, 12, 15, 16). These clinical resemblances may correspond to common biological disturbances, as was recently demonstrated through the experimental generation of impaired N-glycosylation at the Ca_V_2.1 (encoded by *CACNA1A*); impaired N-glycosylation led to a gain-of-function on the Ca_V_2.1 channel (8), similar to those mutations causing FHM (17, 18).

Historically, it has been hypothesized that SLE have a vascular origin, due to the frequent abnormalities in coagulation in CDG patients (13, 19). However, many clinical and neuroradiological features of SLE remain unexplained, while the vascular hypothesis and several symptoms are incongruous.

In this paper, the recent SLE experiences of two PMM2-CDG patients that were exhaustively evaluated during the episodes and who were able to communicate their symptoms are described. These experiences, together with an up-to-date literature review, will provide clinical clues to help improve diagnosis through a proposed clinical constellation of symptoms. A literature review will help to identify evidence-based management recommendations, and the vascular and neuroelectrical hypotheses will be discussed as potential underlying mechanisms in SLE.

## Method

Two patients with confirmed PMM2-CDG and suffering SLE episodes during the previous 24 months were evaluated. The episodes of each fulfilled the following SLE definition: “Acute event consisting of sudden onset of a focal neurological deficit, irritability or decreased consciousness that may associate with seizures, headache or other transient symptoms, in the absence of another diagnosis explaining these symptoms” (8).

Personal direct interview with the patients during the episodes, serial neurological evaluations, laboratory tests, cranial magnetic resonance (MRI) images, and videoelectroencephalogram (video-EEG) were performed during the acute phase of the SLE. MRI was performed with a 1.5 or 3 T scanner (GE Healthcare, Milwaukee, WI, USA or similar). The MRI clinical protocol and sequences included anatomical T1-weighted, T2 weighted fast spin-echo Images, fast-spin echo with fluid-attenuated inversion recovery (FLAIR) coronal, and diffusion weighted images (DWI). VideoEEG recordings were made with plate electrodes fixed on the scalp via “10–20 International System”, and using a digital system with monopolar and differential fittings, which also provides track visualization after recording.

Written informed consent for publication was obtained from the parents and the adult patient, and assent was obtained from the adolescent patient.

For the literature review a search in PubMed for articles on PMM2, PMM2-CDG, CDG-Ia, congenital disorders of glycosylation, and stroke-like as search terms, was performed, from January 1, 1982, and May 29, 2021, with different combinations of the terms.

## Case Reports

### Subject 1

An 11-year-old girl affected by PMM2-CDG (P113L/P113L) was taken to the emergency department for refractory fever up to 39°C of 36 h evolution, along with vomiting and low oral intake. Blood and urine tests were uninformative and the process was cataloged as a viral infectious one causing gastroenteritis.

**Figure 1:**
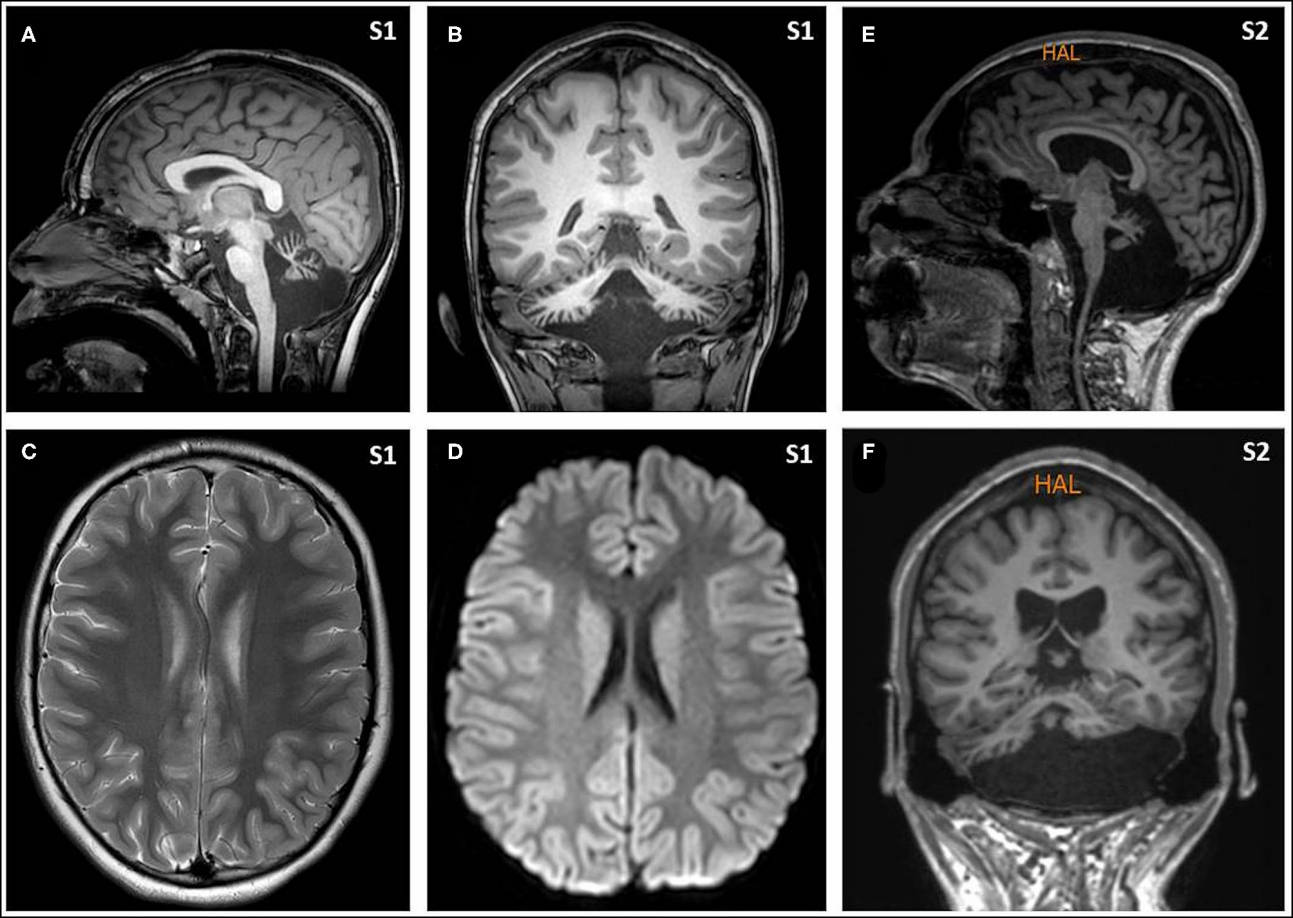
Magnetic resonance imaging (MRI) from Subjects included T1- and T2-weighted images and FLAIR, diffusion weighted images, and an arterial and venous angiographic study, performed with a 3 T and 1.5 T scanners, respectively. There was no abnormal finding other than the cerebellar atrophy. Sagittal [**(A)** from Subject 1, and **(E)** from Subject 2] and coronal images [**(B)** from Subject 1, and **(F)** from Subject 2] show the small cerebellar vermis with enlarged interfolial spaces representing atrophy and secondary enlargement of the fourth ventricle. Axial supratentorial images **(C)**, including diffusion weighted images **(D)**, from Subject 1 show no other abnormalities.

**Figure 2:**
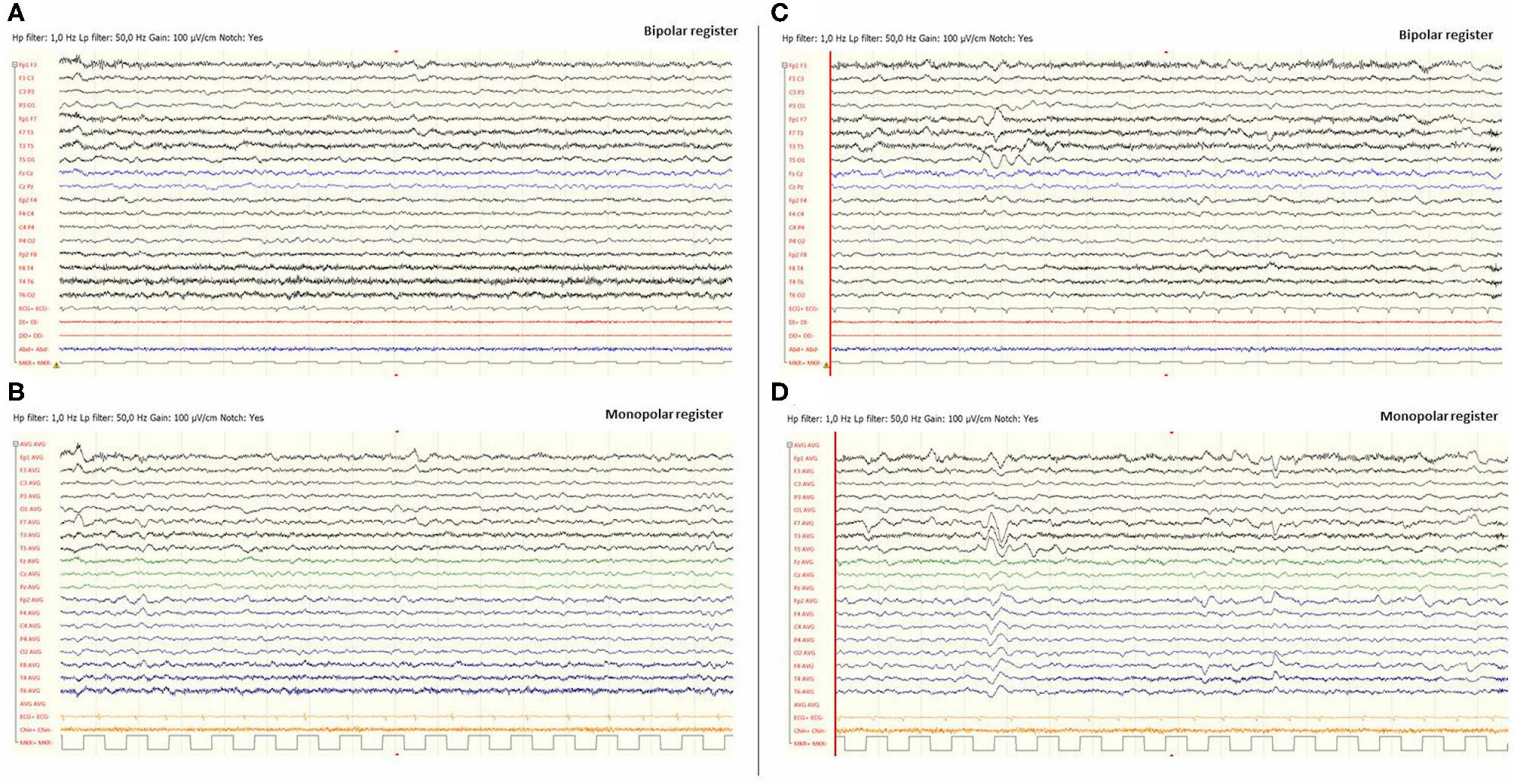
Video-EEG performed during the first episode of Subject 1. EEG was done 12 h after the beginning of the focal deficit and once midazolam had been administered. The EEG was acquired with the eyes open and the patient being awake. EEG showed slow brain activity with subtle asymmetries, delta waves predominantly in the left hemisphere and theta rhythms predominantly in the right [left images **(A)** and **(B)**]. No paroxysms were recorded. Images **(C)** and **(D)**, on the right, include EEG register fragments that show some left temporal intermittent rhythmic delta waves of short duration resembling TIRDA (temporal intermittent rhythmic delta activity).

Eight hours later, despite ibuprofen therapy, the hyperthermia persisted, and the patient suffered a left-sided headache, somnolence, right-arm monoparesis, and particular difficulties in denomination. The patient returned to the emergency department, and, despite suspicion of an SLE, due to febrile neurological symptoms, a ruling-out protocol for treatable disorders was applied. Cranial tomography and MRI scan (Figure 1) were performed, showing no abnormalities on supratentorial structures and demonstrating the expected cerebellar atrophy. Coagulation tests were normal. Lumbar puncture was initially planned but 3 h later, the patient showed improvement, with a normalization of body temperature, increased right arm strength (4/5), normal alertness, and disappearance of the headache. During the next 24 h, the clinical situation oscillated, with limited periods of left-side intense headache associated with coincident worsening of right-arm monoparesis. The MRI obtained at 18 h from the first motor symptoms included T1- and T2-weighted images and FLAIR, DWI, and an arterial and venous angiographic study. There was no abnormal finding other than the cerebellar atrophy. Video-EEG was performed, showing asymmetric slow brain activity with delta waves predominantly in the left hemisphere [see Figure 2 showing left temporal intermittent rhythmic delta waves of short duration resembling TIRDA (temporal intermittent rhythmic delta activity)] and theta rhythms predominantly in the right, as well as low voltage beta rhythms, with a diffuse distribution. No paroxysms were recorded. This episode was the first acute neurological event for this patient.

Fourteen months later, at the age of 13, she suffered a head trauma. Fifteen minutes after the head contusion she showed left arm paresis and incoherent speech followed by a progression of the paresis, reaching the left leg. She began with headache, vomiting, and increasing irritability. At the emergency room she received a total dose of 15 mg of midazolam (5 mg intramuscular, 10 mg intranasal), and a CT scan was performed, showing no supratentorial abnormalities. Six hours after the head trauma, physical exploration was normal, headache had disappeared, and the patient had coherent speech but with slowed thought and response. Eighteen hours after the beginning of SLE the patient was discharged with no abnormal findings compared to her baseline physical exploration. In recent months she has been receiving topiramate (50 mg every 12 h) to control behavioral disturbances. Topiramate was chosen to take advantage of the resemblance of its mechanism of action to that of acetazolamide (20), recently used in a clinical trial for PMM2-CDG (21).

### Subject 2

A 24-year-old young man affected by PMM2-CDG (V44A/R141H) went to the emergency room with intense right-side migraine, propulsive vomiting, photophobia, and, on physical examination, a left hemiparesis. At the outset of the episode with the initiation of the right-side headache, the patient reported a fluctuation of strength on his left side; when he was recovering strength in the lower limb he noted a worsening in the upper limb. No decrease in consciousness or epileptic seizures was detected upon initial examination at the emergency room. However some hours later the patient showed confusion, irritability, and a decrease in alertness. As with Subject 1, lab tests were normal, neuroimaging showed the already known cerebellar atrophy, and video EEG showed right-hemispheric slow brain activity. The full episode lasted approximately 12 hours; the patient was given analgesics (ketorolac and metamizol) and antiemetics (metoclopramide) during the hospitalization.

From infancy the patient had presented with recurrent episodes of somnolence and irritability of several hours' duration, triggered in particular by infection and with associated hyperthermia and epileptic seizures. Antiepileptic treatment with carbamazepine was initiated at 2 years of age and continued until the age of fourteen, after which levetiracetam was prescribed and maintained until the age of eighteen, but it did not seem to prevent SLE recurrence. There was no increase in the rate of occurrence after antiepileptic drug withdrawal. On the contrary, the family reported a subsequent increase in alertness and attention skills.

From adolescence the stroke-like episodes presented with a frequency of one to three times per year and were triggered mainly by heat and dehydration or emotional stress. They were characterized mainly by one-side intense headache with somnolence and irritability, but without epileptic seizures. The fluctuations in strength described in this last episode were previously experienced by the patient and he explained that they occurred for 2–3 h at a time. Headache similarly affected both sides during the different episodes. Motor deficits always presented opposite to the migraine location, and sometimes involved the tongue and mouth movement. The family reported affected speech and denomination when the migraine affected the left side. At the present time the patient is not receiving any pharmacological therapy but the parents limit his exposure to sun and heat and offer him frequent hydrating beverages. He has presented no additional episodes of SLE in the last 18 months.

## Discussion

Clinical situations described in the present report, including experiences of patients in their own voice and exhaustive clinical evaluations, allow us to probe deeper into the phenomenology of SLE, one of the most stressful acute situations for families living with PMM2-CDG.

Remarkably, although it has been reported that up to 50% of PMM2-CDG patients may suffer at least one episode (6, 7), only a few papers on SLE in CDG are indexed in PubMed and there are no cohorts of SLE in PMM2-CDG patients other than a couple of short series (8, 9). Moreover, that SLE is restricted to PMM2-CDG is uncertain, since families of other types of CDG patients share similar episodes as evidenced on social networks and meetings (data unpublished).

SLE are little-known events occurring in a very rare condition, they have been scarcely published and they are probably underdiagnosed. A limitation of the present study is the description of SLE episodes suffered by two patients. However, despite the sample size, this is the first time that a first-person description, including the patients' voice, and the interesting finding of a fluctuation and coincidence of a constellation of symptoms are reported, leading to new mechanistic hypothesis.

SLE are characterized by a confused mental state, focal deficits (mono- or hemiparesis, dysphagia, amaurosis, etc.), hyperthermia, epileptic seizures, and severe encephalopathy in some cases (5, 8–12). Full recovery is expected in most cases in the following days; however, long-term sequelae have also been reported (5, 6, 8, 9, 12).

SLE can present at any age, and seem to occur independently of the clinical global severity (8, 12). Interestingly, recurrent SLE can be the only manifestation of an otherwise asymptomatic child with PMM2-CDG, as has recently been reported (12). Preliminary studies of epidemiological, clinical, laboratory, and neuroimaging findings revealed no differences between PMM2-CDG patients with SLE and those without, and in terms of the genotype, all the pathogenic variants were distributed among both groups (5, 8–12).

In the scientific literature, there are two main trigger factors for SLEs in PMM2-CDG: head trauma and infection (5, 8–12). The role of both conditions in triggering encephalopathic episodes is also described in other inborn errors of metabolism, as well as some channelopathies (14, 15). However the mechanism whereby the mechanical stimulus or the metabolic stress precipitates an SLE is unknown. Although head trauma is a common situation in children with ataxia and hypotonia, due to clumsiness, why only some PMM2-CDG patients develop SLE after cranial trauma is currently unknown.

Unfortunately, there is no specific constellation of clinical symptoms or biomarker that unequivocally characterizes SLE. Clinical, radiological, and electrophysiological findings [including hemispheric EEG diffuse slowing (8–10, 13)] are unspecific and routine lab studies are not informative. Frequently, families suggest the diagnosis to the emergency doctor, so that they can contact the referring doctors, who, however, cannot offer evidence-based clinical guidelines for the management of SLE. Both clinicians and families need accessible and contrasted information.

Due to the absence of positive markers supporting the diagnosis, clinicians are obliged to perform all the tests to rule out treatable disorders, such as central nervous system infections or vascular events, requiring invasive studies such as lumbar puncture and neuroimaging under sedation that may in the end prove unnecessary and sometimes dangerous for the SLE, as has been described (5). Altogether, the scarce scientific evidence and the complex differential diagnosis lead to an absence or a delay in the diagnosis, hindering our knowledge about the real incidence of SLE.

Refractory hyperpyrexia has been described as associated consistently with SLE, probably due to a central origin, as it is often not correlated with inflammatory parameters such as PCT or PCR, or increase in white blood cells (7, 8, 12), and may be refractory to common antipyretics such as ibuprofen and paracetamol (acetaminophen).

The coexistence of migraine, focal neurologic deficits, hemispheric slow EEG trace and refractory hyperpyrexia without laboratory signs of infection make up a characteristic but unspecific tetrad (5, 7–12). In this difficult clinical context, both for diagnosis, but also for the challenging task of increasing scientific knowledge, the use of an accepted clinical definition probes essential (8).

Concerning treatment, there are no evidence-based management guidelines, other than symptomatic measures like antiepileptic drugs, for which concrete publications report an improvement in both seizures and focal deficits (8, 9) similar to that experienced by Subject 1. In this respect, the phenomenology of the SLE described here in which the patients referred a fluctuant headache coincident with a worsening of the motor paresis reinforces the idea of an electric basis for the pathogenesis of SLE and supports the use of antiepileptic drugs. With PMM2-CDG, Dinopoulos' group was the first to indicate the possibility of hemiparesis caused by an active epileptic inhibitory process, supported by EEG findings in three patients during SLE and suggesting, accordingly, anticonvulsant agents for SLE management. The three patients received intravenous lorazepam (0.1–0.2 mg/kg) with an improvement in the EEG recording of one of the patients shortly after administration (9). In our experience, we have seen positive responses to midazolam, as in Subject 1. Although larger samples of patients with SLE in PMM2-CDG are needed to support an evidence-based recommendation, the use of lorazepam or midazolam seems advisable. In addition, the possibility to perform both repeated video-EEG and MRI, from the early detection or suspicion of SLE, before and after the antiepileptic drug administration and later during the SLE resolution, could detect unreported abnormalities and also shed light on the underlying pathophysiology and the best practices for therapeutic management.

As noted above, the term SLE was initially coined for MELAS syndrome (14), stressing its non-ischemic origins. In MELAS, these events are probably explained by an energy failure and the nitric oxide precursor L-arginine seems to exhibit a beneficial effect on the extension, progression, duration, and outcome of a SLE (22). But SLE has also been used for different acute neurological events clinically but not neuroradiologically mimicking an ischemic injury. The events called FHM related to *CACNA1A, ATP1A2*, and *SCN1A* mutations are an example of this (15, 16); clinically they are similar to SLE related to PMM2-CDG and hence, FHM should be in the complex differential diagnosis as reported recently (12). In FHM events it is known that there are underlying electric mechanisms; an inhibitory cortical transmission called “cortical spreading depression” (17). In the FHM murine model, a decreased triggering threshold for cortical spreading depression has been demonstrated (23). In FHM, hemispheric EEG abnormalities, similar to those found in PMM2-CDG SLE (8), have been reported (15, 16). Interestingly, the cortical depression mechanism is also the basis of the migraine pathogenesis (24), explaining the hemispheric electric EEG abnormalities, but also the one-sided headache.

During the acute episodes of SLE and FHM, there are clinical, electrophysiological, and neuroradiological similarities. Episodes in both conditions (i) may be triggered by head traumatisms, (ii) may present after a period free of symptoms, (iii) hyperthermia is frequent (with the absence of any sign of infection in blood or CSF), (iv) abnormal EEG findings may be present, and finally, (v) hemispheric vasogenic edema may occur (8, 15). Interestingly, chronically speaking, clinical, neuroimaging, and neurophysiological features of PMM2-CDG and *CACNA1A* related phenotypes are also similar, including, ataxia, ocular motor disturbances (particularly nystagmus and tonic upgaze deviation), and progressive cerebellar atrophy (3, 8, 25).

Finally, there are also similarities from the pharmacological point of view. Acetazolamide has been used in CACNA1A patients to prevent FHM (26), and a recently designed non-commercial clinical trial using acetazolamide in PMM2-CDG to improve cerebellar syndrome also showed improvement in a treated patient who suffered from almost weekly SLE episodes together with migraine. The patient saw a disappearance of SLE at the beginning of acetazolamide treatment, which returned when the therapy was discontinued following the protocol (21).

In the search for the biological link between PMM2-CDG and FHM, there is some evidence, using experimental generation of impaired N-glycosylation at *CACNA1A* channel and evaluating the subsequent gating abnormalities, that impaired N-glycosylation leads to a gain-of-function on CACNA1A channel, similar to mutations causing FHM (8).

For many years the most widely accepted hypothesis for the pathogenesis of SLE in PMM2-CDG was based on the high frequency of coagulation abnormalities and the very prevalent vascular events (2, 13, 19). However, no significant correlations have been found in a number of studies between the presence of abnormal coagulation and occurrence of SLE (8–12). Furthermore, the majority of patients with SLEs (i) do not show vascular occlusion on magnetic resonance angiography, (ii) have edema images that are not congruent with a cytotoxic edema, but rather a vasogenic edema, (iii) do not follow well-defined vascular territories on brain magnetic resonance images (MRI), and (iv) have lesions that do not reveal restricted diffusion in DWI (signal-intensity changes can be detected within minutes of arterial occlusion with DWI) (8–12). Moreover, the pathomechanisms suggested such as hypoperfusion or ischemia do not explain the nature and temporal course of neuronal dysfunction (8–12).

Despite this lack of evidence, anti-aggregants such as aspirin have been widely used in the attempt to prevent recurrence of SLE, without a scientifically sound basis and running the risk of iatrogenesis while also increasing the risk of developing a bleeding complication. In some patients reported, the use of anti-aggregant doses of aspirin did not prevent the recurrence of SLE (7, 12).

To further complicate matters, from the clinical point of view, the differential diagnosis between SLE, epileptic seizures, a real cerebrovascular event, and a migraine with aura (all prevalent in this group of patients) is a real challenge in PMM2-CDG patients. Moreover, there is a well-studied overlap of biological mechanisms and a known comorbidity among vascular stroke, epilepsy, and migraine (27, 28), since they may share some pathogenic mechanisms.

Head trauma triggering is common not only to channelopathies such as CACNA1A (15, 16), but also to other rare neurological conditions such as vanishing white matter disease (29), and it does not seem to be completely explained merely by a metabolically stressful situation. As occurred in the second SLE of our Subject 1, head trauma is a known trigger for SLE (5, 8, 12), but interestingly, SLE-related neurological symptoms start after a symptom-free interval ranging between one and 24 h (8). Neurons have mechanoreceptors capable of transducing mechanical forces, some of which are N-glycosylated, and abnormalities in N-glycosylation have been proven to alter their functions (30). But after the mechanical stimulus, the symptom-free period reported in these patients points toward the generation of a cascade of changes in the brain until it affects the whole neurological clinical picture, which probably involves the modification of expression of several genes. If this is the case, knowledge of this cascade will discover to us how to treat SLE and prevent progression from a mechanistic point of view.

In conclusion, there is a characteristic but unspecific tetrad of symptoms made up of migraine, focal neurologic deficits, hemispheric slow EEG trace and refractory hyperpyrexia without laboratory signs of infection. However, for clinicians there is an urgent need to positively and unequivocally detect SLE episodes in the group of patients at risk, and based not merely on the ruling out of other acute neurological processes. Invasive studies such as lumbar puncture and neuroimaging under sedation often prove unnecessary and sometimes dangerous for the SLE evolution.

At the present time there is a lack of sound scientific evidence to categorically affirm what the underlying mechanisms beneath SLE are, and therefore evidence-based recommendations for management are very limited, but there is some biological and clinical evidence supporting the use of antiepileptic drugs such as the benzodiazepines lorazepam and midazolam.

Scientific knowledge is still lacking concerning the needs of patients and their families at risk of SLE; a very prevalent and stressful clinical situation that needs to be urgently addressed by researchers.

## Data Availability Statement

The original contributions presented in the study are included in the article/supplementary material, further inquiries can be directed to the corresponding author.

## Ethics Statement

The studies involving human participants were reviewed and approved by CEIC Fundació Sant Joan de Déu. Written informed consent to participate in this study was provided by the participants' legal guardian/next of kin. Written informed consent was obtained from the minor(s)' legal guardian/next of kin for the publication of any potentially identifiable images or data included in this article.

## Author Contributions

MS conceptualized and designed the text, drafted the initial manuscript, and reviewed and revised the manuscript.

## Funding

PMM2-CDG research was supported by National Grants PI14/00021, PI17/00101, and PI21/00068 from the National Plan on I+D+I, cofinanced by ISCIII (Subdirección General de Evaluación y Fomento de la Investigación Sanitaria) and FEDER (Fondo Europeo de Desarrollo Regional). MS research work was supported by a grant from the Generalitat de Catalunya (PERIS SLT008/18/00194).

## Conflict of Interest

The author declares that the research was conducted in the absence of any commercial or financial relationships that could be construed as a potential conflict of interest.

## Publisher's Note

All claims expressed in this article are solely those of the authors and do not necessarily represent those of their affiliated organizations, or those of the publisher, the editors and the reviewers. Any product that may be evaluated in this article, or claim that may be made by its manufacturer, is not guaranteed or endorsed by the publisher.
